# Evaluation of pharmacodynamic biomarkers in a Phase 1a trial of dulanermin (rhApo2L/TRAIL) in patients with advanced tumours

**DOI:** 10.1038/bjc.2011.456

**Published:** 2011-10-27

**Authors:** Y Pan, R Xu, M Peach, C-P Huang, D Branstetter, W Novotny, R S Herbst, S G Eckhardt, P M Holland

**Affiliations:** 1 Department of Molecular Sciences, Amgen Inc., 1201 Amgen Ct. West, Seattle, WA 98119, USA; 2 Department of Pathology, Amgen Inc., 1201 Amgen Ct. West, Seattle, WA 98119, USA; 3 Genentech, South San Francisco, CA, USA; 4 Yale Medical Oncology, Smilow Cancer Hospital at Yale-New Haven, South Frontage Road and Park Street, 4th Floor, New Haven, CT 06510; 5 University of Colorado Cancer Center, 12801 East 17th Avenue, Aurora, CO 80045, USA; 6 Department of Oncology, Amgen Inc., 1201 Amgen Ct. West, Seattle, WA 98119, USA

**Keywords:** Apo2L/TRAIL, dulanermin, biomarker, Phase 1a trial, caspase, M30

## Abstract

**Background:**

Dulanermin (rhApo2L/TRAIL) induces apoptosis by binding to death receptors DR4 and DR5, leading to caspase activation and subsequent cell death. A Phase1a trial evaluated the safety and tolerability of dulanermin in patients with advanced tumours. One aim was to develop and validate pharmacodynamic biomarkers to monitor dulanermin activity in patient serum.

**Methods:**

We optimised assays to measure the cell-death markers caspase 3/7, cytokeratin 18 and genomic DNA in serum. Mice bearing Colo205 xenografts were treated with dulanermin and sera were collected and assayed for apoptotic markers. Upon validating these assays, we monitored apoptotic markers in patients who received dulanermin.

**Results:**

We detected transient increases in apoptotic markers in mouse sera 8–24 h after dulanermin treatment. This increase was dose-dependent and correlated with active caspase 3 detected by IHC in Colo205 tumours. A statistically significant increase in serum caspase 3/7 was detected in cohorts of colorectal and sarcoma patients 24 h after receiving dulanermin dosed above 4 mg kg^−1^.

**Conclusion:**

Owing to limited responses in the Phase 1a study, the changes in circulating cell-death markers were not evaluable. Future studies with dulanermin are needed to determine the utility of these assays with respect to providing evidence of activity or predicting overall response.

Tumour cell evasion of apoptosis is fundamental to the pathogenesis and progression of cancer, and strategies to develop apoptosis-based therapies in cancer represent a potential new treatment paradigm that may be applicable to a wide range of tumour types (Fesik, 2005). Since cancer cells are under stress and highly dependent on aberrations of apoptosis signalling pathways to survive, selective killing of tumour cells might be achievable with apoptosis-promoting drugs. One apoptosis-inducing agent being explored for cancer therapy is dulanermin, an optimised soluble form of the endogenous Apo2 ligand/TNF-related apoptosis-inducing ligand (Apo2L/TRAIL). Apo2 ligand/TNF-related apoptosis-inducing ligand is a member of the TNF superfamily that induces apoptosis through engagement of death receptors. Apo2 ligand/TNF-related apoptosis-inducing ligand binds to two death receptors, DR4 (TR-1) and DR5 (TR-2), that contain cytoplasmic death domains and initiate an apoptosis signalling cascade (Pitti *et al*, 1996; Pan *et al*, 1997; Sheridan *et al*, 1997; Walczak *et al*, 1997). This results in the activation of caspases, cysteine proteases that cleave intracellular substrates, leading to cellular apoptosis. Apo2 ligand/TNF-related apoptosis-inducing ligand also binds three additional receptors, DcR1, DcR2 and OPG, which do not trigger apoptosis upon ligand engagement (Degli-Esposti *et al*, 1997a, 1997b; Marsters *et al*, 1997; Sheridan *et al*, 1997; Emery *et al*, 1998). Early functional studies indicated that Apo2L/TRAIL has a potent ability to preferentially trigger apoptosis in a variety of tumour cell lines *vs* normal cells, highlighting its potential as a candidate therapeutic in cancer (Wiley *et al*, 1995; Pitti *et al*, 1996). Dulanermin exhibits single-agent anti-tumour activity as well as cooperativity with various conventional and targeted agents in tumour xenograft models (Ashkenazi *et al*, 2008). Early clinical trial data also suggested that dulanermin was generally safe and well tolerated and provided preliminary evidence of anti-tumour activity (Herbst *et al*, 2010).

Based on the pre-clinical data, the spectrum of tumours that may respond to dulanermin therapy is broad. Therefore, the application of informative, validated biomarkers of apoptosis in the clinical development life cycle could be important in the selection of indication, dose, real-time monitoring of the biological activity of the drug and possibly prediction of clinical outcome. A pharmacodynamic (PD) end point may be defined as a biological effect that is a measure of altered activity or expression of a molecular target in response to a mechanism-based therapy (Sarker and Workman, 2007). Pharmacodynamic markers for cell death include measurement of active caspase 3/7, caspase cleaved cytokeratins and circulating DNA fragments (Ward *et al*, 2008). Caspase activation has been used as a marker for the detection of apoptotic cells before they are engulfed by phagocytes (Durrieu *et al*, 1998). Cytokeratins are important components of the intermediate filaments of epithelial cells and are insoluble under normal physiological conditions. During cellular necrosis, release of soluble intact cytokeratin 18 (CK18) can be detected (Stigbrand, 2001). During cellular apoptosis, the intermediate filaments, including CK18, are targeted for rapid breakdown by activated caspases 3, 7 and 9 to facilitate the formation of apoptotic bodies (Kramer *et al*, 2004). A comparison of the relative amounts of cleaved *vs* uncleaved circulating CK18 can therefore distinguish between necrosis and apoptosis as the modes of cell death (Linder, 2007). Cytokeratin 18 and its proteolytic fragments are stable and can be detected in the circulation of cancer patients using commercially available immunoassays (Ku *et al*, 1997; Biven *et al*, 2003). The observation that cancer patients have elevated levels of circulating DNA compared to healthy individuals has also led to the application of monitoring serum DNA fragments as a surrogate for disease as well as response to therapy (Leon *et al*, 1977). Thus, the biological products of apoptosis may be utilised not only to monitor pathological states such as cancer, but also to provide insight on the activities of therapeutic agents in various disease settings (Ulukaya *et al*, 2011).

We evaluated whether circulating increases in PD markers of cell death could be detected in response to dulanermin treatment in a tumour xenograft model. The primary circulating apoptotic biomarkers in our study included active caspase 3/7 and caspase-cleaved CK18. Dulanermin induced a transient dose-dependent increase in circulating caspase 3/7 and cleaved CK18 in the Colo205 xenograft tumour model, and these increases correlated with positive caspase 3/7 activity on tumour biopsies by immunohistochemistry. These findings provided rationale for the evaluation of clinical samples from the Phase 1a trial for dulanermin. In colorectal (CRC) and sarcoma patients, there was a statistically significant increase in circulating caspase 3/7 activity following dulanermin administration. We also observed a trend towards increased circulating cell-death biomarkers in a number of non-small-cell lung cancer (NSCLC) patients. Our results support the utility of measuring serum caspase 3/7 and cleaved CK18 as potential PD biomarkers to monitor dulanermin activity in patients with advanced solid tumours.

## Materials and methods

### Reagents and assays

Dulanermin (rhApo2L/TRAIL) was produced at Genentech Inc. (South San Francisco, CA, USA). Caspase 3/7 activity was determined using an optimised and qualified Promega Caspase-Glo 3/7 assay (Promega Corp., Madison, WI, USA). Samples were diluted in Caspase-Glo 3/7 assay buffer (human serum to 15%, mouse serum to 25%) and mixed 1 : 1 with Caspase-Glo 3/7 assay substrate, for 90 min at 30 °C. Luminescence was measured and reported as CPS (PerkinElmer EnVision Model 2102 plate reader, Waltham, MA, USA) or RLU (Molecular Devices Analyst HT, Sunnyvale, CA, USA). To control for inter-assay variation, raw data values from clinical samples were normalised to the value obtained with a normal, human positive control (human serum with endogenous caspase 3/7) and reported as % of control. Haemolysed samples were disqualified based on visual inspection. Caspase-cleaved and full-length CK18 were detected using the M30-Apoptosense and M65 ELISA assays, respectively (Peviva AB, Stockholm, Sweden). Recombinant human active caspase-3 was from Biomol International (Plymouth Meeting, PA, USA). The caspase 3/7 inhibitor, Ac-DEVD-CHO, was from Promega. Genomic DNA (gDNA) was extracted from human serum using the Chemagic Viral DNA/RNA kit (Chemagen, Portland, ME, USA) and subjected to real-time qPCR analysis (Taqman assay, ABI Prism 7900HT instrument; Applied Biosystems, Carlsbad, CA, USA) for amplification of human *β*-actin using the following primer and probe sequences: forward primer, 5′-TCCTCCTGAGCGCAAGTACTC-3′ reverse primer, 5′-CTGCTTGCTGATCCACATCTG-3′ and probe, 5′-6-FAM-ATCCTGGCCTCGCTGT-MGB-3′.

The final amount of gDNA in the sample was determined by interpolation from a standard curve of known quantities of commercially available gDNA (Becton Dickinson, Franklin Lakes, NJ, USA).

### Subcutaneous xenografts

**Figure 1:**
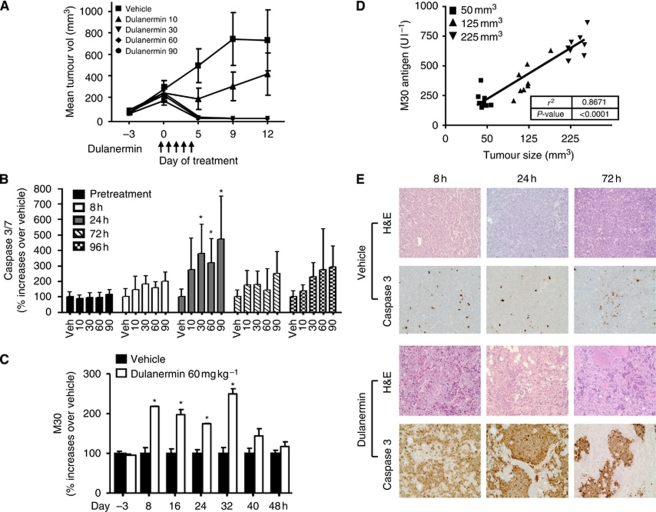
Effect of dulanermin-induced pharmacodynamic marker increases in a Colo205 xenograft model. (**A**) Average tumour size in Colo205 xenografts treated with dulanermin. Mice were injected subcutaneously with 7 × 10^5^ Colo205 cells (*n*=10 per group). When tumours reached approximately 200 mm^3^, mice were treated with either phosphate-buffered saline (PBS) or dulanermin (10, 30, 60 and 90 mg kg^−1^ intraperitoneally on days 1–5). Tumour volumes were monitored over the treatment period and up to 12 days and are expressed as mean±SD. (**B**) Serum samples from Colo205 tumour-bearing mice (treated as described in **A**) were collected at the indicated times and analysed using the Promega Caspase Glo assay (*n*=7 per group). Data were initially plotted as mean±SD for each group and then converted to % of change over the vehicle group at each time point. Significant per cent increases in caspase 3/7 activity were seen at 24 h in the three higher dose groups as compared with vehicle control (30 mg kg^−1^, *P*=0.0089 *vs* vehicle; 60 mg kg^−1^, *P*=0.0112 *vs* vehicle; 90 mg kg^−1^, *P*=0.0129 *vs* vehicle). (**C**) Colo205 tumour-bearing mice (as described in **A**) were treated with either 60 mg kg^−1^ dulanermin or PBS daily for 2 days. Serial serum samples were collected at the indicated times and analysed using the M30 Apoptosense ELISA assay (day −3 (pretreatment)=18 per group; all other time points=6 per group). Data were initially plotted as mean±SD for each group and then converted to % of change over the vehicle group at each time point. M30 values were extrapolated by the plate reader through the standard curve. ^*^P<0.0001 *vs* vehicle at each time point. (**D**) Mice were inoculated with either 3 × 10^5^, 5 × 10^5^ or 1 × 10^6^ Colo205 cells and treated with one dose of 60 mg kg^−1^ dulanermin when tumours reached approximately 50, 125 or 225 mm^3^ (*n*=8 per group). Serum samples were collected before treatment and 24 h after treatment and analysed using the M30 Apoptosense ELISA assay. (**E**) Dulanermin-induced Colo205 tumour-specific caspase 3 activity correlates with circulating serum caspase 3 and M30 antigen levels. Histological sections from Colo205 tumours in mice receiving either vehicle or dulanermin (60 mg kg^−1^) were collected at 8, 24 or 72 h and stained with haematoxylin and eosin (H&E) or active caspase 3. Images are × 20 magnification.

Colo205 and HT29 cells (ATCC, Manassas, VA, USA) were cultured in RPMI 1640 (Invitrogen, Carlsbad, CA, USA) supplemented with 10% FCS (HyClone Laboratories, Logan, UT, USA), L-glutamine and antibiotics. Female CB17 SCID mice (Charles River Laboratory, Wilmington, MA, USA) aged 8–10 weeks were injected subcutaneously with 7 × 10^5^ parental Colo205 cells (*n*=10 per group) or 1 × 10^6^ HT29 cells (*n*=5 per group). When tumours reached 150–200 mm^3^, mice were treated with vehicle (PBS) or dulanermin (10, 30, 60 and 90 mg kg^−1^ intraperitoneally daily for 5 days, *n*=10 per group) or as described. Tumour dimensions were measured by a digital calipre and tumour volumes were calculated as (length × (width)^2^)/2. For the reduced tumour volume study shown in Figure 1D, mice were inoculated with either 3 × 10^5^, 5 × 10^5^ or 1 × 10^6^ Colo205 cells and treatment initiated when tumours reached approximately 50, 125 or 225 mm^3^ (*n*=8 per group). Tumour volumes are expressed as mean±s.d. For serum collections, all mice were serially bled via tail nick in the lateral tail vein. All mice underwent a pretreatment bleed, and groups of mice were staggered such that no mice were bled more than once every 24 h. Serum was not pooled for analysis. All experimental procedures were under the Amgen animal use guidelines and approved by the Amgen Institutional Animal Care and Use Committee.

### Immunohistochemistry

Cross-sections of each tumour were collected in 10% neutral buffered formalin, fixed and embedded in paraffin. Sections were stained with haematoxylin and eosin or for cleaved caspase 3. Caspase 3 staining was performed as described previously (Lippa *et al*, 2007).

### Patient sera collection for PD analysis

Whole blood (8.5 ml) was collected into Vacutainer (Becton Dickinson) tubes at specified time points. Serum was separated from cells by centrifugation at 1500 g at 4 °C for 15 min and stored at −70 °C until the time of the assay.

### Statistical analyses

For changes in serum caspase 3/7 activity and M30 levels in mice with Colo205 xenografts, a repeated measure ANOVA model was fitted on the proportional change using PRCO GLIMMIX of SAS 9.1 (SAS Institute, Cary, NC, USA). The covariance structures ANTE(1) was used to model residual covariance matrix. The Dunnett's method was used for multiple comparison adjustment. For clinical sample analysis, Wilcoxon's signed-rank test was used to evaluate the median % of change of cell-death biomarkers from pretreatment baseline at various time points. For dosing correlation, the Jonckheere–Terpstra trend test was used to detect whether a per cent change in cell-death biomarker level at a given time point varied significantly across ordered dose levels, followed by the Cochran–Armitage trend test. The Kruskal–Wallis test was used to explore the association between per cent change from baseline of cell-death biomarkers at each time point and best tumour response. Finally, an exploratory multivariable regression model of the log ratio of caspase 3/7 activity level at 24 h to baseline level was fit to evaluate the contribution of dosing, best response and tumour type.

## Results

### Pre-clinical validation of apoptosis markers in the serum

The aim of our studies was to determine if markers of dulanermin-induced apoptosis could be monitored in the serum. To this end, we focused on two related molecular end points, caspase 3/7 activity and detection of the caspase 3 substrate CK18. To determine whether any markers of dulanermin-induced apoptosis could be monitored in the serum, we utilised the Colo205 xenograft model, which we have previously characterised for sensitivity to dulanermin *in vitro* and *in vivo* (Lippa *et al*, 2007). We compared the efficacy of 10, 30, 60 or 90 mg kg^−1^ dulanermin administration in the Colo205 xenograft model. With the exception of the 10 mg kg^−1^ group, all doses resulted in full tumour regressions by day 5 of treatment (Figure 1A). During the course of 5-day dulanermin treatment, we examined the levels of circulating active caspase 3/7 in the serum at 8, 24, 72 and 96 h. The 8 and 24 h collections occurred after the first dose; the 72 and 96 h collections were 24 h after the third and fourth dose, respectively. Although there was no significant increase in serum caspase 3/7 activity in mice 8 h after dulanermin treatment, serum caspase 3/7 activity was significantly increased at 24 h in the three higher dose groups as compared to vehicle control (30 mg kg^−1^, *P*=0.0089 *vs* vehicle; 60 mg kg^−1^, *P*=0.0112 *vs* vehicle; 90 mg kg^−1^, *P*=0.0129 *vs* vehicle) (Figure 1B). These data indicate that significant serum caspase 3/7 elevations correlated with doses that resulted in tumour regressions. The average serum caspase 3/7 activity remained elevated relative to vehicle at 72 and 96 h in the three higher dose groups, but the difference was not statistically significant, potentially due to the larger within-group variation at those time points (Figure 1B). In an independent experiment, we also evaluated the circulating levels of caspase-3 cleaved CK18 using the M30 ELISA assay. In this analysis, we focused only on the 60 mg kg^−1^ dose of dulanermin, which has been previously demonstrated to provide optimal anti-tumour activity in pre-clinical models and resulted in tumour regressions (Ashkenazi *et al*, 1999; Kelley *et al*, 2001), and evaluated additional time points around 24 h, where significant serum caspase 3/7 increases were observed. Figure 1C shows that M30 antigen levels were elevated as early as 8 h after dulanermin treatment. The comparisons at individual post-dose time points indicated that M30 antigen levels were significantly increased from 8 to 32 h in the dulanermin-treated groups compared to vehicle (*P*<0.0001). There was no significant difference at 40 or 48 h. Collectively, these data indicate that in response to dulanermin treatment, serum-based apoptosis markers show a transient increase that peak around 24 h and gradually decline thereafter.

To determine whether the magnitude of caspase 3/7 cleaved CK18 corresponded with tumour burden, we compared circulating M30 antigen levels from dulanermin-treated mice (60 mg kg^−1^) with established Colo205 tumours of variable size (either 50, 125 or 225 mm^3^ at time of dosing) 24 h after dosing. Figure 1D indicates that there was a significant positive correlation between circulating M30 antigen levels and tumour size following treatment with dulanermin.

To determine how changes in serum-based apoptosis markers corresponded to changes within the tumour, we examined caspase 3/7 activity in Colo205 tumours by immunohistochemistry. As shown in Figure 1E, treatment of Colo205 tumour-bearing mice with 60 mg kg^−1^ dulanermin resulted in a robust increase in tumour caspase 3 staining that was readily detectable at 8 and 24 h, and began to decrease by 72 h. Thus, the kinetics of dulanermin-mediated caspase activation in the tumour parallel the appearance of cell-death markers (active caspase 3/7, cleaved CK18) in the serum and suggests that the circulating markers are tumour derived and occur in response to dulanermin treatment. Consistent with this, we did not detect dulanermin-dependent increases in circulating cell-death markers in either mice bearing dulanermin-resistant tumourous or non-tumour-bearing mice (Supplementary Figure 1). Although we noticed a high background signal of the M30 antigen in subsequent studies on mouse serum as noted in other publications (Cummings *et al*, 2008), the batches of reagent used for this study were from earlier lots and did not require additional blocking, probably due to batch-to-batch variation of the mouse antibody reagents (personal communication with Peviva). No background with the M30 antigen was observed on human samples.

### Assay validation for human sample analysis

**Figure 2:**
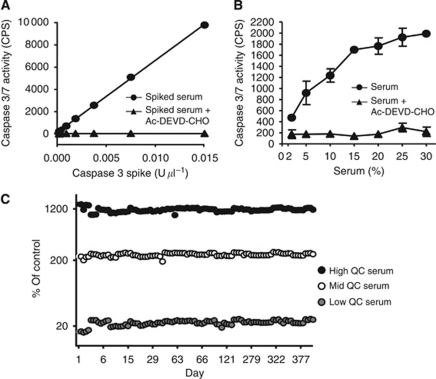
Validation for human serum caspase 3/7 assay specificity, linearity and reproducibility. (**A**) Recombinant human caspase 3 was spiked into 15% normal human serum in the presence or absence of 25 *μ*m caspase 3/7 inhibitor Ac-DEVD-CHO and luminescence was measured. (**B**) Caspase 3/7 activity was measured in human serum samples diluted 2–30% in Caspase-Glo 3/7 buffer in the presence or absence of the 25 *μ*m caspase 3/7 inhibitor Ac-DEVD-CHO. Serum dilution linearity was only observed in the dilutions below 15%. (**C**) Three serum controls containing various levels of caspase 3/7 activity were analysed repeatedly every 2–3 weeks over the course of 14 months. Samples were stored at −70 °C between repeat analyses.

Our analysis of the circulating cell-death markers caspase 3/7 and M30 antigen in dulanermin-treated Colo205 tumour-bearing mice provided proof-of-principle that these analytes show transient increases in the serum following dulanermin administration, which correlate with dulanermin-mediated anti-tumour activity. To extend these types of analyses to clinical samples, we first undertook a rigorous process of assay validation. Use of the M30 and M65 ELISA assays with clinical samples has been established previously (Olofsson *et al*, 2007). For the caspase 3/7 activity assay and gDNA assay, our validation criteria included assay sensitivity, precision, specificity, range, sample stability and intra-donor variation. For example, to demonstrate the specificity of the caspase 3/7 assay, recombinant caspase 3 was spiked into 15% normal human serum and luminescence was measured in the presence or absence of the caspase 3/7 inhibitor Ac-DEVD-CHO. As caspases 3 and 7 recognise the same substrate, we did not run the assay with caspase 7. Figure 2A shows that a linear relationship of luminescent signal to spiked recombinant caspase 3 was observed, which was completely blocked in the presence of the caspase inhibitor, showing that the signal was specific for caspase 3. To define assay linearity, human serum was diluted to various concentrations with Caspase-Glo 3/7 buffer and caspase 3/7 activity was measured in the presence or absence of Ac-DEVD-CHO. Assay linearity was only observed in the serum dilutions below 15% (Figure 2B). Based on these findings, human serum samples were diluted to 15% in Caspase-Glo 3/7 buffer on all subsequent analyses. Under this assay condition, the level of detection of this assay was defined as 1.12% control serum. The control serum was run along with all samples including the clinical samples. The assay was qualified as a quasi-quantitative assay (Lee *et al*, 2005). To assess the performance of the caspase 3/7 activity assay, three human serum samples with various levels of endogenous caspase 3/7 activity were analysed repeatedly every 2–3 weeks over the course of 14 months and included to run along with clinical samples. The assay performed consistently in qualification and production with inter-plate % CV of <14.1% (Figure 2C). A similar assay validation was conducted for the M30, M65 and gDNA assays (data not shown). For the gDNA assay, the average CV over an 18-month period was below 7.16% (data not shown). To evaluate intra-patient variation, three blood draws were collected sequentially from six healthy volunteers at four possible time points (0, 24, 48 and 168 h) and serum was analysed for caspase 3/7 activity. Within each donor, mean caspase 3/7 values were consistent over the time points evaluated, with an average CV of 13.3% (Supplementary Table 1).

### PD marker analysis of dulanermin Phase 1a clinical study samples

The dulanermin Phase 1 clinical trial examined the safety, tolerability and maximum-tolerated dose (MTD) of multiple doses of dulanermin when administered to subjects with advanced or metastatic solid tumours or non-Hodgkin's lymphoma (Herbst *et al*, 2010). Additional objectives were to characterise the pharmacokinetics of dulanermin, to make preliminary assessments of dulanermin's anti-tumour activity and to assess the utility of exploratory biomarkers. This study was conducted in two stages that included the same treatment and monitoring schedule in parallel for subjects with normal liver enzyme levels and without liver metastases (Cohort 1) and for subjects with liver metastases with either normal liver enzyme levels or mildly abnormal liver function (Cohort 2). The doses of dulanermin evaluated were 0.5, 1.5, 4.0, 8.0, 15.0, 20.0 and 30.0 mg kg^−1^, administered by intravenous infusion over 1 h on each of days 1–5 of a 21-day cycle for up to 24 weeks. There were 72 patients enrolled and 71 treated. There were 13 tumour types represented, and the most frequent primary tumour types were: CRC (14 (20%)), NSCLC (9 (13%)) and sarcoma (9 (13%)). Overall, dulanermin was well tolerated and an MTD was not reached. The majority of patients (56%) exhibited stable disease and two patients with chondrosarcoma showed a partial response (Herbst *et al*, 2010).

Serum samples for exploratory biomarker analyses were collected at baseline, 5, 24 and 504 h (21 days) after dulanermin treatment. These times corresponded with subject availability for sample collection and also with our pre-clinical observations with respect to peak levels of circulating cell-death markers. Owing to concerns over patient welfare and protocol guidelines, we were not able to obtain multiple pre-dose samples. Of the 71 subjects treated, 56 subjects had baseline caspase 3/7 data. Of the 56 patients with baseline caspase 3/7, 46 had data at 5 h, 52 at 24 h and 41 at 504 h. There were 62 patients with baseline gDNA data; of these, 57 had gDNA data at 5 h, 62 at 24 h and 49 at 504 h. It is worth noting that not all subjects with baseline samples had samples collected at all subsequent time points; therefore, the analyses were limited to the availability of paired samples from baseline and the time points indicated. In terms of the overall patient cell-death biomarker responses, there was a significant trend between increased dose of dulanermin and occurrence of any increase in circulating caspase 3/7 and gDNA levels 5 h after treatment (Table 1). However, owing to the small sample size for the majority of tumour types, our analyses focused on those that were best represented in the trial, namely CRC, NSCLC and sarcoma.

**Figure 3:**
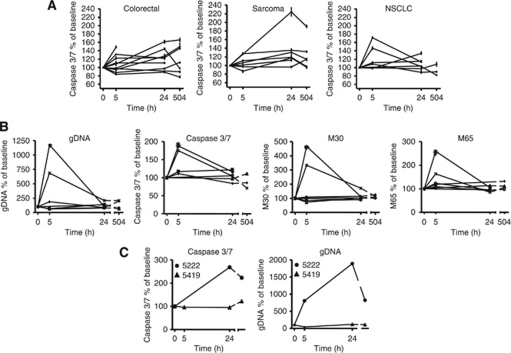
Changes in circulating biomarker levels in tumour subgroups. (**A**) Percentage caspase 3/7 activity over baseline in colorectal (CRC), sarcoma and non-small-cell lung cancer (NSCLC) patient serum samples collected at 5, 24 and 504 h post dulanermin treatment is indicated. The mean of un-normalised triplicates at each time point is used for the calculation and each line represents an individual patient. Error bars in graphs show the min and max ratio based on all possible pairs of the numerator and denominator triplicates. (**B**) Comparison of cell-death marker analytes in NSCLC subjects. Values depicted were determined as described above. (**C**) Comparison of circulating caspase 3/7 activity and genomic DNA (gDNA) levels in chondrosarcoma subjects 5222 and 5419. Both subjects showed a partial response to dulanermin treatment. Subject 5222 (30 mg kg^−1^ dose group) had a 124 and 90% increase in serum caspase 3/7 at 24 and 504 h, respectively (5 h time point was missing), and a 704, 1788 and 723% increase in gDNA levels at 5, 24 and 504 h, respectively. Subject 5419 (8 mg kg^−1^ dose group) had a −3, −3 and 15% change in serum caspase 3/7 at 5, 24 and 504 h, respectively, and a −56, 18 and 10% change in gDNA levels at 5, 24 and 504 h, respectively.

At 5 h, no significant trend of increased serum caspase 3/7 was observed in the specific tumour types; the median per cent change from baseline caspase 3/7 levels was 3% for CRC, 12% for NSCLC and 1% for sarcoma patients (Figure 3A and Table 2). At 24 h after dulanermin administration, there was a statistically significant increase in circulating caspase 3/7 levels from CRC (median increase: 23%, *P*=0.04) and sarcoma patients (median increase: 19%, *P*=0.03) (Figure 3A and Table 2). At 24 h, five of eight (63%) CRC patients and three of seven (43%) sarcoma patients had at least a 20% increase in circulating serum caspase 3/7 from baseline. No significant serum caspase 3/7 increase was seen at 24 h for the NSCLC patient samples analysed. However, in two of seven NSCLC patients with evaluable biomarker measurements at baseline, a significant increase in the magnitude of all four cell-death biomarkers was observed 5 h after dulanermin administration (Figure 3B). Owing to the small sample size, the ratio of M30/M65 antigen was not evaluated. The trend towards an increase in circulating cell-death biomarker levels at 5 h in the NSCLC patient samples may reflect a difference in the kinetics of apoptosis induction in NSCLC *vs* CRC and sarcoma patients. In CRC patients, increases in cleaved CK18 at 24 h post dulanermin treatment relative to baseline were also observed (*P*=0.03, data not shown). Owing to the non-epithelial origin of sarcomas, these tumours do not express CK18. Therefore, only the serum caspase 3/7 assay was used to evaluate samples from sarcoma patients. Within the CRC and sarcoma subjects, there was no correlation between mean per cent change in circulating caspase 3/7 from baseline and dose group or tumour response (Table 3). This was confirmed by the multivariate regression analysis. Interestingly, one of the two chondrosarcoma patients that showed a partial response also exhibited the highest increase from baseline in both circulating caspase 3/7 and gDNA levels 24 h after dulanermin administration (Figure 3C). There was no difference in changes in cell-death analytes between cohorts 1 (CRC patients with no liver metastasis) and 2 (CRC patients with liver metastasis), nor was there any correlation with the number of distant metastatic sites. Additional analyses were conducted to assess correlations between circulating apoptosis markers and changes in markers of liver function, namely aspartate aminotransferase (AST) and alanine aminotransferase; however, the sample size was too small to draw any conclusions (data not shown). Also, owing to the small sample size and exploratory nature of these biomarkers, multiple testing adjustment across biomarkers was not carried out. A subsequent and properly powered Phase 2 study would be needed to validate these findings.

## Discussion

Here we report that circulating biomarkers of apoptosis show a transient increase in response to dulanermin therapy in a pre-clinical model, and this increase correlates with tumour cell death. We qualified an assay for measuring serum caspase 3/7 levels from clinical samples and found that circulating caspase 3/7 increased in a statistically significant manner in CRC and sarcoma patients treated with dulanermin. Our study is the first report where cell-death PD markers have been used to assess the evidence of biological activity in patients treated with dulanermin monotherapy.

As the number of pro-apoptotic therapies in pre-clinical and clinical development increases, there is a need for validated biochemical coverage markers that report apoptotic cell death in accessible tissues such as the serum. Reports are emerging of changes in circulating biomarkers of cell death in response to apoptosis targeted therapies. For example, treatment of mice bearing H146 lung cancer xenografts with the Bcl-2 inhibitor ABT-737 leads to transient increases in circulating CK18 and cleaved caspase levels that correlate with treatment and tumour burden (Micha *et al*, 2008). Cytokeratin 18 increases have also been reported in response to doxorubicin treatment of FaDu head and neck carcinoma xenografts, and following treatment of SW620 colon carcinoma xenografts with the Aurora kinase inhibitor AZD1152 (Cummings *et al*, 2008; Olofsson *et al*, 2009).

Preliminary analysis of circulating cell-death markers to monitor anti-tumour responses in a clinical study has also been reported. In a Phase 1 trial of the XIAP antisense inhibitor AEG35156, 28 of 38 patients exhibited significantly elevated biomarker responses (M30/M65 CK18 plasma levels), which were temporally associated with drug infusion (Dean *et al*, 2009). No responses greater than stable disease were reported in this study, and in at least one patient these responses tracked with transient AST levels, indicative of hepatotoxicity.

In our study, we observed a trend towards increased circulating serum caspase 3/7 and gDNA levels in patients treated with increasing doses of dulanermin. We also observed a statistically significant increase in serum caspase 3/7 activity levels in CRC and sarcoma patients 24 h post dulanermin treatment in the first treatment cycles, suggesting a biological effect induced by dulanermin in cancer patients. The levels of serum caspase 3/7 significantly increased in response to dulanermin treatment in CRC patients, suggesting dulanermin-mediated apoptosis induction in these subjects. A confounding factor in our findings may be that only one pretreatment sample was available for baseline analysis, and therefore would not adequately account for intra-patient variation. Of note, the biological variations in our intra-patient analysis did not reflect any particular directional trend, in contrast to the trend observed following dulanermin treatment in certain cohorts. Changes in markers measuring total cell death, namely circulating total CK18 (as measured by M65 ELISA) and gDNA levels, were less consistent. With the exception of a subset of NSCLC patients at the 5 h time point, there was no correlation between caspase 3/7 activity and total CK18 or gDNA levels. The increase in caspase 3/7 activity occurred earlier in NSCLC patients than in CRC and sarcoma patients. Although our study is limited by the small sample size in each of the three main tumour subtypes evaluated, it is possible that the kinetics of apoptosis induction may vary in tumour subtypes, and the performance of these cell-death markers may be tumour-type dependent.

Several questions are raised by some discrepancies in our pre-clinical and clinical findings. First of all, in contrast to our pre-clinical results, the clinical analysis showed little correlation between cell-death PD markers and tumour response. While one of the two partial response patients with sarcoma histology demonstrated a large increase in caspase 3/7 activity and circulating gDNA 24 h post-treatment, most of the patients with induced serum caspase 3/7 activity in this study had progressive disease in spite of dulanermin treatment. One possible explanation for the lack of correlation between PD marker and response is that sustained pathway activation by dulanermin is necessary for achieving a durable clinical response. Because cell-death markers were only analysed in the first cycle of treatment in the clinical study, we cannot draw any conclusions about the effects of dulanermin in subsequent cycles. It is likely that circulating cell-death biomarkers also have a temporal component. For example, previous studies have demonstrated that M30 and M65 levels correlate with established testicular cancer tumour markers and tend to be low in testicular cancer patients who have a good prognosis, possibly indicative of low tumour burden (de Haas *et al*, 2008). Moreover, in patients who have a complete response, M30 levels show a transient increase following chemotherapy treatment and then decrease to below pretreatment levels at the end of the study, suggestive of a decrease in overall tumour load over time. If sustained pathway activation following dulanermin treatment is required, early PD marker measurements as performed in this study would not be predictive of overall response. Alternatively, PD marker assessment over the full course of treatment as a marker of drug activity at each cycle, or at the end of treatment as a marker of changes in overall tumour burden, may be more informative in this regard.

Secondly, we cannot determine from our clinical results whether a particular threshold or magnitude of caspase activation must be reached or sustained to result in a durable anti-tumour response. The magnitude of increase in serum caspase 3/7 activity in clinical samples was modest compared with that observed in the pre-clinical xenograft model. This could be due to a difference in sensitivity to dulanermin in primary human tumours. Alternatively, the differences in relative tumour burden between the pre-clinical model and patients, or a difference in the clearance mechanism of circulating analytes by macrophage engulfment, may also play a role. Although it is possible that a correlation between cell-death marker increases and overall response may not be expected, this would not have been predicted based on our pre-clinical findings.

Lastly, because we measured analytes in circulation, it remains unclear whether the increased circulating serum caspase 3/7 detected in CRC and sarcoma patients originated from tumour or from other tissues. It is possible that secondary effects of the malignancy or drug toxicity resulted in spurious serum caspase 3/7 elevations in some patients, although the safety profile was consistent across all patients in the study (Herbst *et al*, 2010). Circulating M30 antigen has been shown to be a marker of host tissue toxicity in sepsis and liver disease (Roth *et al*, 2004; Yilmaz *et al*, 2009). Ideally, measuring caspase 3/7 activation directly from tumour samples post-treatment would be the preferred approach for assessing biochemical coverage of the pathway. Whereas caspase 3/7 activity on repeated fine-needle aspirate biopsies in a clinical study has been reported, successful implementation of this approach remains challenging (Zoog *et al*, 2010). Based on our pre-clinical and clinical findings regarding peak circulating cell-death biomarker elevations, and in consideration of patient availability and convenience, we would select 24-h post-treatment as a time point to assess apoptosis in future trials with dulanermin.

In summary, our pre-clinical findings and qualified serum caspase 3/7 assay development provide proof-of-principle for the use of cell-death biomarker assays to monitor dulanermin activity in a clinical setting. We found significant increases in the levels of circulating caspase 3/7 in cohorts of CRC and sarcoma patients treated with dulanermin. However, owing to the small number of evaluable patients and limited responses in the dulanermin Phase 1a study, the changes in circulating cell-death markers were not predictive. Data from future clinical studies with dulanermin should help to determine the utility of these assays with respect to providing evidence of activity or predicting overall response.

## Figures and Tables

**Table 1:** Summary of serum caspase 3/7 and gDNA changes in subjects by dose group

**Dulanermin**	**Per cent of subjects with caspase 3/7 increase (total *n*)**		**Per cent of subjects with gDNA increase (total *n*)**	
**(mg kg^−1^)**	5 h*	**24 h**	5 h**	**24 h**
1.5	20 (1/5)	40 (2/5)	0 (0/5)	20 (1/5)
4	33 (2/6)	75 (6/8)	25 (2/8)	56 (5/9)
8	50 (11/22)	56 (14/25)	48 (13/27)	59 (17/29)
15	80 (4/5)	83 (5/6)	83 (5/6)	75 (6/8)
30	NA	NA	80 (4/5)	60 (3/5)

Abbreviations: gDNA=genomic DNA; NA=not applicable.

Table shows per cent and number of patients from all tumour types showing any increase in circulating caspase 3/7 and gDNA by dose group. Dose groups containing <5 evaluable patients were not included. Cochran–Armitage trend test *P*-values indicating a trend of dose-dependent increase in serum caspase 3/7 and gDNA in circulation after 5 h (only *P*-values <0.05 are shown).

^*^P=0.0412.

^**^P=0.0061.

**Table 2:** Median per cent change and range of serum caspase 3/7 over baseline level in CRC, sarcoma and NSCLC subjects

	**Colorectal**	**Sarcoma**	**NSCLC**
*5 h*			
*N*	10	5	5
Median % change (range)	3 (−16, 49)	1 (−13, 27)	12 (−2, 72)
*P*-value^*^	0.43	0.81	0.13
			
*24 h*			
*N*	**8**	**7**	7
Median % change (range)	**23 (−11, 62)**	**19 (−3, 124)**	4 (−12, 34)
*P*-value^*^	**0.04**	**0.03**	0.22
			
*504 h*			
*N*	7	6	3
Median % change (range)	12 (−23, 66)	15 (−4, 90)	−10 (−18, 8)
*P*-value^*^	0.30	0.16	

Abbreviations: CRC=colorectal; gDNA=genomic DNA; NSCLC=non-small-cell lung cancer.

Data associated with significant *P-*values are shown in bold.

^*^Wilcoxon signed-rank test *P*-value indicating a trend of increased caspase 3/7 from baseline to the time point within the tumour type (only *P*-values <0.10 are shown). With the exception of NSCLC at 5 h, there was no correlation with caspase 3/7 activity and M65 or gDNA levels.

**Table 3:** Mean per cent change in caspase 3/7 levels from baseline listed for CRC and sarcoma subjects by dose group and tumour response

**Diagnosis**	**Dose (mg kg^−1^)**	**Best response**	**PFS (weeks)**	**Median % change caspase 3/7 (0–24 h)**
Colorectal	1.5	PD	5.3	−2.5
Colorectal	1.5	PD	5.4	44.7
Colorectal	4	SD	24.1	−10.7
Colorectal	4	SD	11.3	10.9
Colorectal	8	PD	1.4	21.7
Colorectal	8	PD	5.1	27.5
Colorectal	8	PD	5.3	61.7
Colorectal	15	PD	5.1	23.3
Synovial sarcoma	8	PD	3.1	12.7
Hemangiosarcoma	8	Unknown	0.1	17.4
Angiosarcoma	8	SD	5.1	35.5
Chondrosarcoma^a^	8	PR	79.1	−3.2
Chondrosarcoma	8	SD	12.3	30.6
Chondrosarcoma	30	Unknown	0.1	18.5
Chondrosarcoma^a^	30	PR	12.3	124.0

Abbreviations: CRC=colorectal; gDNA=genomic DNA; PD= progressive disease; PR=partial response; PFS=progression free survival; SD=stable disease.

a Corresponds to extended treatment subjects. With the exception of NSCLC at 5 h, there was no correlation with caspase 3/7 activity and M65 or gDNA levels.
