# Supplementary material for: Evaluation of pharmacodynamic biomarkers in a Phase 1a trial of dulanermin (rhApo2L/TRAIL) in patients with advanced tumours
Source: Br J Cancer. 2011 Oct 27;105(12):1830–8. doi: 10.1038/bjc.2011.456 (PMC3251880; doi:10.1038/bjc.2011.456)
Supplement: Supplementary Information [file bjc2011456x1.ppt]

## Slide 1
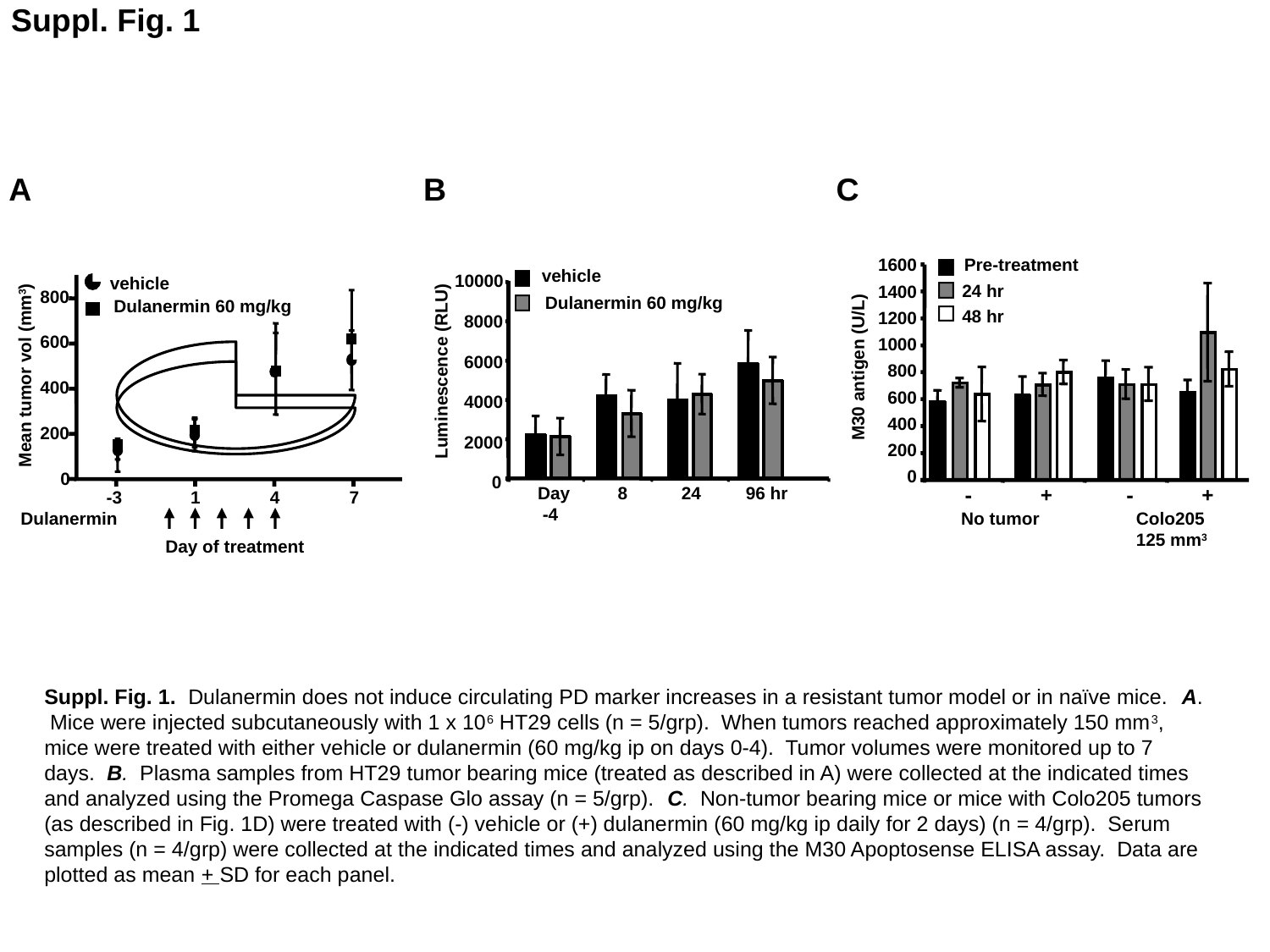

Suppl. Fig. 1
A
B
C
1600
Pre-treatment
24 hr
1400
48 hr
1200
1000
M30 antigen (U/L)
800
600
400
200
0
-
+
-
+
No tumor
Colo205
125 mm3
vehicle
10000
Dulanermin 60 mg/kg
8000
6000
Luminescence (RLU)
4000
2000
0
Day
 -4
8
24
96 hr
vehicle
800
Dulanermin 60 mg/kg
600
Mean tumor vol (mm3)
400
200
0
-3
1
4
7
Dulanermin
Day of treatment
Suppl. Fig. 1. Dulanermin does not induce circulating PD marker increases in a resistant tumor model or in naïve mice. A. Mice were injected subcutaneously with 1 x 10^6^ HT29 cells (n = 5/grp). When tumors reached approximately 150 mm3, mice were treated with either vehicle or dulanermin (60 mg/kg ip on days 0-4). Tumor volumes were monitored up to 7 days. B. Plasma samples from HT29 tumor bearing mice (treated as described in A) were collected at the indicated times and analyzed using the Promega Caspase Glo assay (n = 5/grp). C. Non-tumor bearing mice or mice with Colo205 tumors (as described in Fig. 1D) were treated with (-) vehicle or (+) dulanermin (60 mg/kg ip daily for 2 days) (n = 4/grp). Serum samples (n = 4/grp) were collected at the indicated times and analyzed using the M30 Apoptosense ELISA assay. Data are plotted as mean + SD for each panel.

## Slide 2
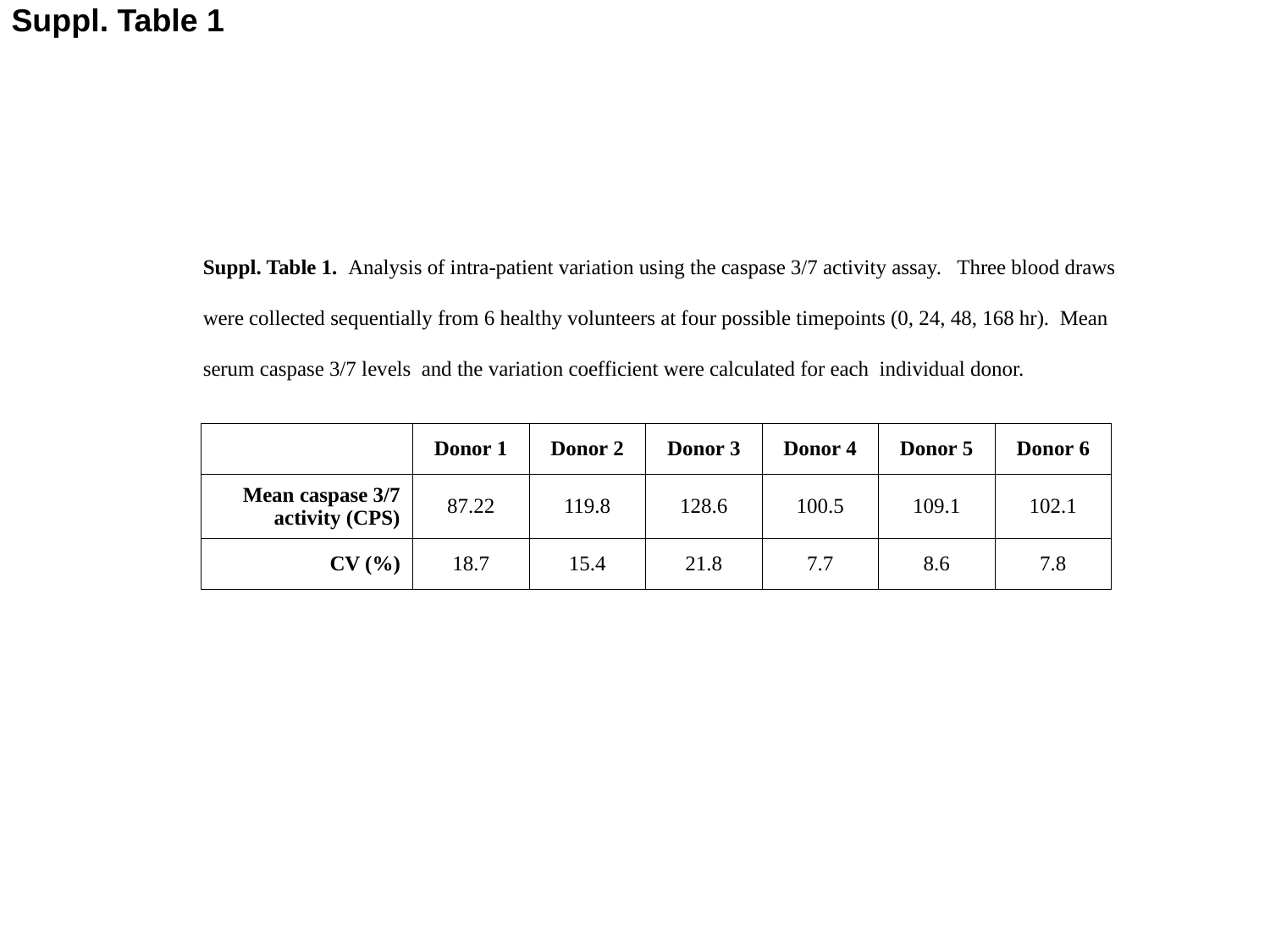

Suppl. Table 1
Suppl. Table 1. Analysis of intra-patient variation using the caspase 3/7 activity assay. Three blood draws were collected sequentially from 6 healthy volunteers at four possible timepoints (0, 24, 48, 168 hr). Mean serum caspase 3/7 levels and the variation coefficient were calculated for each individual donor.
| | Donor 1 | Donor 2 | Donor 3 | Donor 4 | Donor 5 | Donor 6 |
| --- | --- | --- | --- | --- | --- | --- |
| Mean caspase 3/7 activity (CPS) | 87.22 | 119.8 | 128.6 | 100.5 | 109.1 | 102.1 |
| CV (%) | 18.7 | 15.4 | 21.8 | 7.7 | 8.6 | 7.8 |
